# Recommencement of Sport Leagues With Spectators at the Adelaide Oval During the COVID-19 Pandemic: Planning, Experience, and Impact of a Globally Unprecedented Approach

**DOI:** 10.3389/fpubh.2021.676843

**Published:** 2021-07-23

**Authors:** Joel Ern Zher Chan, Angela Lee, Chris Lease, Nicola Spurrier

**Affiliations:** ^1^Health Regulation and Protection, Department for Health and Wellbeing, Adelaide, SA, Australia; ^2^Adelaide Medical School, Faculty of Health and Medical Sciences, University of Adelaide, Adelaide, SA, Australia; ^3^Infectious Diseases Unit, Royal Adelaide Hospital, Adelaide, SA, Australia; ^4^College of Medicine and Public Health, Flinders University, Bedford Park, SA, Australia; ^5^Paediatric General Clinic, Flinders Medical Centre, Bedford Park, SA, Australia

**Keywords:** COVID-19, sport event, mass gathering events, sport leagues, stadiums, non-pharmaceutic measures, risk assessment, health legacy

## Abstract

Non-pharmaceutical interventions including physical distancing and restriction on public gatherings were the cornerstone in controlling the COVID-19 pandemic, in the absence of effective vaccines and available treatment options. Many sport mega-events and sport leagues were canceled or indefinitely postponed, leaving stadiums globally empty or converted to be used as part of the COVID-19 response. There were calls for exit strategies to be developed. With the early containment of COVID-19 in South Australia, various restrictions were lifted in a staged and controlled manner, including the reopening of the Adelaide Oval for recommencement of sport leagues with spectator attendance. This involved the collaboration between public health authorities, other governmental agencies, Adelaide Oval Stadium Management Authority, various sporting leagues etc. Recommencement of sport leagues with staged increase in number of attending spectators allowed various measures to be introduced, revisited, and implemented accordingly, demonstrating that a case-by-case risk assessment can be conducted for mega-events during COVID-19, accounting for the epidemiological context at the time. Economic impacts and non-economic benefits of this recommencement were documented. This globally unprecedented, staged and controlled approach in returning spectators to sporting events during the COVID-19 pandemic could inform the reopening strategy of stadiums, recommencement of sport leagues and mega-events all over the world before herd immunity is achieved or in the event of future outbreaks.

## Introduction

Since its initial outbreak in Wuhan, China (1), COVID-19 has rapidly spread throughout the world. As of July 2020, there had been more than ten million COVID-19 cases and more than five hundred thousand deaths globally (2). In the absence of a vaccine and available treatment options, control of COVID-19 relied on non-pharmaceutical interventions, including physical distancing measures, rapid case identification and isolation, contact tracing and quarantine of close contacts, restriction on public gatherings, cancellation of public events, and border closures (3, 4); leaving global impacts on employment, socio-economy, sports industry, hospitality and tourism, and many other areas (5). They also left many stadiums around the world empty, with stadiums in the more affected countries converted into field hospitals, testing sites, homeless shelter, morgues etc. (6).

Sporting activities have well-documented roles in primary, secondary and tertiary prevention of chronic physical diseases (7, 8) and psychological well-being (9–12). Encouraging physical activity had been an important public health intervention, prompting health authorities to encourage ongoing physical activity during the pandemic (13, 14). The sports industry further confers economic and non-economic benefits both for individuals and for the society (15–18). A recent study interestingly found a correlation between subjective life satisfaction among sport spectators who spectated a sport match at stadiums, but not when watching broadcasted sport matches (19).

In the midst of a global pandemic, sporting activities and mega-events play an important role in maintaining physical health and mental well-being, but the approach to these needed to prevent and mitigate the risks of COVID-19 transmission into and within the community. For this reason, most sporting events scheduled for 2020 were either canceled, suspended or postponed, including the Tokyo 2020 Olympics and Paralympics, Union of European Football Associations Euro 2020 football championship, 2019-2020 National Basketball Association season, etc. (20).

These cancellations, suspensions and postponements were, however, almost unprecedented (20). Sport mega-events and sport leagues have previously proceeded despite Public Health Emergencies of International Concern, including the Vancouver 2010 Olympics during the pandemic influenza A H1N1, Rio 2016 Olympics during the Zika virus outbreak, and various African sport mega-events including the Africa Cup of Nations during the 2013-2016 Ebola virus disease epidemic (21, 22).

On the other hand, some evidence suggests that these were due to evidence-based or foreseeable reduced impact of such events despite the epidemiological situation at that time (23), in contrast to the ongoing acceleration of COVID-19 (2). A sport mega-event had also been implicated as one of Thailand's main source of COVID-19 (24, 25).

On balance of the various benefits of the sports industry both to individuals and to societies, historical precedence, as well as forward planning, there have been calls for planning and preparation of sports mega-events and sport leagues to commence, and for the use of risk assessments to guide this process (26–28), although there had also been concerns that such calls were premature and a reflection of complacency, as physical distancing and continued disinfection of hard surfaces were “impossible” during mega-events (29).

Evidently, recommencement of sport leagues and sport events had implications for individual health and well-being as well as public health, but needed to take into account potential community risks and for appropriate mitigating measures to be in place during the pandemic.

## Context

In South Australia, a Public Health Emergency was declared in response to COVID-19 on March 15, 2020 under the *South Australian Public Health Act 2011*, and a Major Emergency under the *Emergency Management Act 2004* on March 22. Various restrictions implemented included a mandatory 14 day-quarantine for all people arriving from outside of South Australia, closure of various premises and activities including indoor and outdoor sporting venues as well as social sporting-based activities and competitions, limiting the maximum number of people present at these venues and activities, physical distancing measures and density requirements to determine the maximum number of patrons at various premises, etc. These impacted sport leagues and sporting venues including the Australian Football League (AFL), the South Australian National Football League (SANFL), and the Adelaide Oval.

AFL is the professional competition and governing body for Australian rules football, the most popular sport attended and watched via broadcast in Australia (30–32). An oval ball or the “footy” is moved across an oval field through various techniques like kicking, handballing, or running, with the aim of kicking it between two central goal posts, scoring the team six points. The team scores one point if the ball passes between one of these central goal posts and an adjacent external post called “behind,” or if the ball hits a goal post.

Comprising of 18 teams from five different Australian states, AFL matches take place in various stadiums throughout Australia. Home games for the two South Australian football clubs, the Adelaide Football Club and the Port Adelaide Football Club, are typically hosted at the Adelaide Oval, including the Showdown matches where these two clubs play against each other. Shortly after commencing its 2020 Toyota AFL Premiership season on March 12, AFL announced on March 13 that most remaining matches would proceed without spectators for the foreseeable future, and that there was potential interruption across the season. The womens' league was canceled for the year and the men's season was postponed on March 22.

Australian rules football is also played at other levels of competition, with SANFL as South Australia's football league and governing body for the sport. All SANFL teams are based in South Australia, with its matches typically hosted in suburban grounds throughout Adelaide. During the COVID-19 pandemic, SANFL also delayed the start of its 2020 State League.

The Adelaide Oval is managed by the Adelaide Oval Stadium Management Authority (AOSMA). It has a seating capacity of 50,083 in its grandstands with a record crowd number for a sporting event of 55,317 during the Second Ashes Test: Australia vs. England in 2017 (33). There are also function rooms at the Adelaide Oval, typically used as corporate facilities during major events at the Adelaide Oval.

Following a prolonged period of no active COVID-19 cases, various restrictions that were previously implemented were lifted in a staged and controlled manner (Supplementary Figure 1), including restrictions concerning indoor and outdoor sports training and competition, restrictions around hospitality, maximum number of people at these venues and activities etc.

## Key Programmatic Elements

### Epidemiological Context

Planning for the staged and controlled recommencement of sport leagues and reopening of the Adelaide Oval commenced in May 2020. At the time South Australia had 438 cases of COVID-19, with the last case being notified to the Department for Health and Wellbeing (SA Health) on April 21, 2020. Most of these cases acquired the infection overseas or from a close contact, and there was a limited number of cases acquired locally in South Australia without a close contact being successfully identified. The epidemiology of COVID-19 remained well-controlled in South Australia as the planning continued and sport leagues resumed, with only five further cases being notified as of 1 July 2020. All five cases were among returning travelers from overseas or had established close contact with a confirmed COVID-19 case. All these cases were required by law to quarantine in a designated facility for 14 days following arrival in South Australia, effectively isolated from the general South Australian population (34). This epidemiological context is informed by the existing statewide surveillance system, which included an expanded testing capacity initiated as part of the COVID-19 emergency response, as well as mandatory requirement for all confirmed COVID-19 cases to be notified to SA Health by pathology providers and diagnosing clinicians.

### Collaborative Planning: Initial Meetings and Recommencement Plans

The staged and controlled reopening of the Adelaide Oval and recommencement of sport leagues was planned with a collaborative approach among key stakeholders, including public health authorities, other governmental agencies, AOSMA, AFL, and SANFL (Table 1).

**Table 1:** Key stakeholders and their roles in the reopening of Adelaide Oval and recommencement of sport leagues in South Australia.

	**Agency**	**Role**
Governmental agencies	Department for Health and Wellbeing (SA Health)	• Ensure sport leagues and sporting venues are aware of public health principles that enable the safe return of sporting activities and associated activities. • Along with other governmental agencies (including Department for Premier and Cabinet and South Australian Police), ensure that proposed plans comply with legislative requirements and are adequate to prevent transmission of COVID-19 should the plan proceeds. Exemptions are provided as appropriate. • Undertake site visits before and during sport events. • Provide support to sport leagues and sporting venues as required, including health advice, appropriate resources, key messages for targeted communications, feedback and upskilling support. • Encourage downloading of contact tracing mobile application. • Respond to any intentional or unintentional breach in guidelines and restrictions. • Provide feedback to sport leagues and sporting venues. • Share learnings with other Australian jurisdictions via the Australian Health Protection Principal Committee. • Consistent messaging to the general public and targeted communications prior to events.
	South Australian Police (SAPOL)	• Along with other governmental agencies (including SA Health and Department for Premier and Cabinet), ensure that proposed plans comply with legislative requirements. Exemptions are provided as appropriate and in discussion with SA Health. • Undertake site visits before events and liaise with event organizers to identify areas requiring support. • Provide support for road closures, crowd management, security and safety both within sporting venues and surrounding areas. • Respond to any intentional or unintentional breach in guidelines and restrictions. • Provide feedback to sport leagues and sporting venues. • Consistent messaging to the general public and targeted communications prior to events.
	Department for Premier and Cabinet (DPC)	• Along with other governmental agencies (including SA Health and South Australian Police), ensure that proposed plans comply with legislative requirements. Exemptions are provided as appropriate and in discussion with SA Health and SAPOL. • Consistent messaging to the general public and targeted communications prior to events.
	Office for Recreation, Sport and Racing (ORSR)	• Ensure sport leagues and sporting venues are aware of public health principles that enable the safe return of sporting activities and associated activities. • Work closely with sport leagues and liaise closely with SA Health as required. • Consistent messaging to the general public and targeted communications prior to events.
	Department for Infrastructure and Transport (DIT)	• Provide support for road closures, alternative access and additional public transport overlay as required. • Provide information to sport spectators and general public on road closures, alternative access and changes to public transport. • Liaise with local government councils to facilitate additional car parking options.
Non-governmental agencies	Adelaide Oval Stadium Management Authority (AOSMA)	• Liaise closely with governmental agencies to ensure management plans for events at the Adelaide Oval comply with legislative requirements and public health principles. • Identify areas where support is required and liaise with governmental agencies as required. • Ensure venue is set up according to proposed plan, including clear markings on ground, audiovisual reminder, adequate staffing etc. • Consistent messaging to the general public and targeted communications prior to events. • Encourage downloading of contact tracing mobile application. • Respond to any intentional or unintentional breach in guidelines and restrictions. • Seek feedback from attending spectators; provide feedback and share learnings with governmental agencies. • Consistent messaging to the general public and targeted communications prior to events. • Continue to collaborate with other key stakeholders in planning for the next event.
	Sport leagues including AFL and SANFL	• Liaise closely with governmental agencies to ensure plans for sport events comply with legislative requirements and public health principles. • Ensure league officials and elite athletes comply with proposed plans. • Respond to any intentional or unintentional breach in guidelines and restrictions. • Consistent messaging to the general public and targeted communications prior to events.

Online meetings began in May, involving the Chief Public Health Officer (NS) and Deputy Chief Public Health Officer (CL), Minister for Sports, Office for Recreation, Sport and Racing and representatives from all major sporting organizations in South Australia. Public health principles that enable the safe return of various sporting activities and prevent community risks were explained, allowing for representatives from various sporting organizations to ask any questions. Activities often carried out in conjunction to sporting activities, e.g., sales of food and beverages during sports training and sporting matches, were also discussed. Sporting organizations were referred to the Australian Institute of Sports Framework to reboot sport in a COVID-19 environment (35); which provides principles on how sporting activities can safely resume, and was endorsed by the National Cabinet of Australia.

Office for Recreation, Sport and Racing worked closely with individual sporting organizations on their individual plans for recommencement of sporting activities and was in regular consultation with SA Health. Where these plans required additional consideration or exemption from restrictions at the time, consultation and approval were sought from SA Health, Department for Premier and Cabinet and South Australian Police (SAPOL).

AFL and SANFL's protocols for the recommencement of training and matches were provided to SA Health for feedback and endorsement, as well as to the AOSMA to allow planning of the Adelaide Oval logistics and operations in line with their protocols. The AOSMA strategic reopening plan was also provided to SA Health and SAPOL for feedback and endorsement.

Site inspections were undertaken at the Adelaide Oval prior to each match, involving a walk-through of the Adelaide Oval and discussions on the various aspects of the reopening strategy. The timing of each site inspection allowed for sufficient time to implement changes.

### Fixture and Rule Changes; Training and Competition Arrangements

The AFL fixture was reduced to 17 rounds, with all 18 teams playing each other once throughout the season. The fixture was also released gradually to allow for regular changes at short notice where necessary. Each match was played with shortened quarters and time on. Given the 18 teams being based at five different states, a consistent start date for non-contact and contact training was negotiated between AFL and public health authorities of the states separately. Alternative arrangements, including relocation of teams to another jurisdiction, were made as required. A COVID-19 protocol including physical distancing measures and regular COVID-19 testing was proposed with strict adherence required of officials, staff, players, coaches, and where necessary, members of their household.

As a number of Australian states had ongoing quarantine requirements for travelers from another jurisdiction at the time of planning and recommencement of sport leagues, this presented an additional consideration for AFL when organizing their fixtures. To facilitate a modified fixture for its 2020 season that complied with state border restrictions at the time, it was proposed for some football clubs to be accommodated in hubs located in another jurisdiction, where they train and compete.

AFL officials entering South Australia from states with quarantine requirements were required to undertake 2 weeks of quarantine prior to the match, with exemptions considered on a case-by-case basis. Initial AFL matches in South Australia only involved teams that had been in jurisdictions with no quarantine requirements. Subsequently, quarantine of international elite athletes and athletes who had been in epidemiologically high-risk jurisdictions were facilitated for other sport leagues, with training and maintenance of physical fitness allowed via carefully considered corridors from the site of quarantine to the site(s) of training and physical activities, without exposure to the wider community.

### Staged and Controlled Reopening of the Adelaide Oval

**Figure 1:**
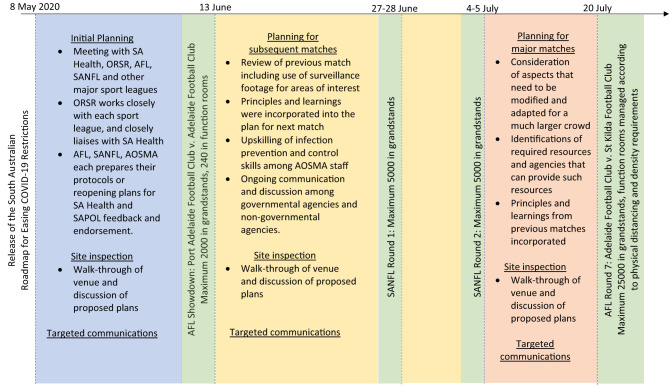
Timeline illustrating the staged and controlled recommencement of sport leagues with spectator attendance at the Adelaide Oval. SA Health, Department for Health and Wellbeing; ORSR, Office for Recreation, Sport and Racing; AFL, Australian Football League; SANFL, South Australian National Football League; AOSMA, Adelaide Oval Stadium Management Authority; SAPOL, South Australian Police.

The number of spectators allowed in attendance at the Adelaide Oval was progressively increased, beginning with an approval for a crowd of 2,000 spectators, followed by 5,000 spectators for several matches, and subsequently 25,000 spectators (Figure 1). These represented 11, 14, and 50% seating capacity of the utilized grandstands at the Adelaide Oval respectively. The relatively small percentages of seating capacity as well as allocated seating in an approved seating configuration allowed for effective physical distancing during the matches.

Additional spectators were hosted in separate function rooms according to the density requirement and physical distancing guidelines during the time of each match. Each function room was served by its own kitchen and staff, and food and beverage in individual portions.

Spectators from other jurisdictions were able to attend matches at the Adelaide Oval when there were no border closures to the jurisdictions in which they reside. All spectators were required to enter their contact details and answer an online declaration form when downloading their mobile tickets.

Various measures were also put in place to mitigate frequent, random and spontaneous interactions, with considerations also made for car parking, public transport, spectator ingress and egress, vertical transport, toilets, food and beverage facilities, merchandise, general cleaning, hygiene and waste management. These mitigating measures are described in detail and are available as Supplementary Material 1. These mitigating measures were introduced, revisited, and implemented according to the increasing number of spectators. Resources including staffing were provided for the maximum capacity in each separate section of the Adelaide Oval.

### Learnings, Upskilling, and Sharing of Information

Observations from each match were noted. Minor improvements were implemented on the night, while others were taken on notice and improved upon after the match. Representatives from Office for Recreation, Sport and Racing and major sport organizations in South Australia were invited for a walk-through on the night of the first match. This allowed for mitigating measures implemented at the Adelaide Oval to be observed by the representatives, to inform the recommencement of sport training, competition and associated activities in their respective organizations.

Surveillance footage for areas of interest were reviewed by AOSMA in conjunction with SA Health after the match. Support for upskilling of infection prevention and control measures among AOSMA staff was provided by SA Health and these measures were implemented in the subsequent matches.

Noted observations and suggestions for improvement were also provided to the Australian Health Protection Principal Committee, the key decision-making committee for health emergencies in Australia. The committee comprises of Chief Health Officers from all Australian jurisdictions as well as subject matter experts. Following their consideration of this matter, a statement on the safe return of crowds to stadiums, arena and large theaters was released by the Australian Health Protection Principal Committee (36).

### Documenting the Impact of COVID-19, Reopening of Adelaide Oval and Recommencement of Sport Leagues With Spectator Attendance

Media reports from major news outlet in South Australia and official releases were searched for relevant reports between March 1 and July 27, 2020. These dates represented 2 weeks prior to the recommencement of the AFL season, and 1 week after the AFL match with an approved maximum of 25,000 spectators. Social media results which were not reported by traditional media or official websites were excluded. Included search outcomes were qualitatively analyzed using a bottom up, inductive thematic analysis approach (37). The aim of this analysis was not to develop generalizable truths for all sport leagues, mega-events or sporting venues, but to develop meaningful insights into the impact of COVID-19, the reopening of Adelaide Oval and the recommencement of sport leagues with spectator attendance. The findings of this analysis are available as Supplementary Material 2 and their implications discussed below.

## Discussion

To the authors' best knowledge, this Australian approach of a staged and controlled reopening of stadiums to allow recommencement of sport leagues with spectator attendance is the first of its kind during the global COVID-19 pandemic. With the COVID-19 pandemic continuing to impact on most countries around the world, it is hoped that the approach taken and the findings in the reopening of the Adelaide Oval and recommencement of sporting leagues and sport mega-events in Australia could inform the reopening strategy of stadiums, recommencement of sporting leagues and sport mega-events all over the world, considering the local epidemiology and community risks.

### Practical Implications

The most important practical implication of this local experience in reopening of the Adelaide Oval and recommencement of sporting leagues and sport mega-events in Australia is the demonstration that recommendations and considerations for the planning and operationalization of sport mega-events is indeed possible and can be safely carried out, with no new cases of COVID-19 resulting as a result of this public health intervention.

Media analysis around the staged and controlled reopening of the Adelaide Oval and recommencement of AFL and SANFL with spectator attendance confirmed the previous findings of the sports industry conferring economic impacts as well as non-economic benefits, including enhancement of individuals' sense of well-being, reinforcement of community cohesion, community visibility, and image (15–17).

The satisfaction reported by South Australian sport spectators during COVID-19, including those whose teams lost in the matches they spectated, along with the positive media narrative around the reopening of Adelaide Oval and recommencement of AFL and SANFL could be seen as supporting the finding of Oh et al. (19). This could present a further argument for a case-by-case risk assessment for sport mega-events with spectator attendance during the COVID-19 pandemic (27, 28), provided that public health measures including physical distancing, hygiene as well as infection prevention and control measures could be adequately implemented.

The considerations as part of this risk assessment (27, 38) are described as follows.

#### Normative and Epidemiological Context

The epidemiological context in South Australia during this public health initiative could be described as “no reported cases” under the World Health Organization's four COVID-19 transmission scenarios, with occasional “sporadic cases” effectively separated from the rest of the South Australian community (34, 38, 39).

The reopening of Adelaide Oval and recommencement of AFL and SANFL with spectator attendance also reflected the normative context of COVID-19 restrictions previously implemented being lifted in a staged and controlled manner. The reopening of Adelaide Oval and recommencement of AFL and SANFL in South Australia complied with restrictions and SA Health recommendations at the time, with only certain exemptions provided by SA Health and SAPOL following satisfactory planning and discussions with AOSMA, AFL, SANFL, and other stakeholders.

#### Evaluation of Risk Factors

The Adelaide Oval is considered an outdoor venue. The staged and controlled reopening of Adelaide Oval allowed for a progressive increase in the maximum number of spectators of 2,000, 5,000, and 25,000, representing 11, 14, and 50% seating capacity of the utilized grandstands. While frequent, random and spontaneous interactions could be expected at typical football matches, various measures were put in place to mitigate this. The duration of each match had been shortened following the COVID-19 pandemic.

The capacity of the health system to detect and manage cases of COVID-19 was also adequate at the time of planning and operationalization of these matches, including expanded testing capacity that had been initiated as part of the COVID-19 emergency response and the requirement for COVID-19 cases to be notified by pathology providers and diagnosing clinicians.

#### Capacity to Apply Prevention and Control Measures

These relatively small percentage of seating capacity as well as allocated seating in an approved seating configuration allowed for physical distancing measures to be effectively practiced during the matches. Additional spectators were hosted in function rooms according to the statewide density requirement and physical distancing guidelines. Groups of spectators were hosted in separate function rooms, each served by its own kitchen and staff, and food and beverage served in individual portions. Resources including staffing were provided for the maximum capacity in each separate section of the Adelaide Oval.

Physical distancing, hygiene, and infection prevention and control measures were possible and observed to be satisfactory during ingress and egress (including security check and admission), vertical transport, and other elements around the matches (including purchase of food and beverage, toilets, etc.). Physical distancing was managed by having clear signage, ground markings to facilitate physically distanced queues and visual reminders, well-positioned staff, security surveillance and back-up crowd control measures. Encouraging spectators to present early reduced congestion and further enabled physical distancing. Other measures to encourage crowd flow include SAPOL presence at potential congestion points and road closures where required.

The expectation for spectators to be seated during most of the matches greatly reduced the contact rate between spectators who are not of the same household. Furthermore, the potential for attending spectators to be infectious with COVID-19 were minimized by the epidemiological context in South Australia, ongoing border closure to jurisdictions of high risk for community transmission, existing legislations requiring returning travelers, confirmed cases, and close contacts to be in isolation or quarantine, and requiring spectators to electronically declare an absence of COVID-19 symptoms before being directed to download their mobile tickets. The use of mobile tickets and allocated seating also meant accurate details for attending spectators were available for contact tracing should the need arose.

#### Determination of the Overall Risk

Overall, the risk of transmission and further spread of COVID-19 was considered very low, taking into account the normative and epidemiological context, evaluation of risk factors, and the capacity to apply prevention and control measures. Ongoing public health risk assessments also occur prior to each match with close liaison between all key stakeholders.

### Lessons Learnt for Future Application

A key enabling factor in the reopening of Adelaide Oval and recommencement of sport leagues and mega-events is the early collaborative planning among key stakeholders, both from government agencies and non-government agencies. There was a shared understanding of the public health situation in South Australia, the need for ongoing measures, and the impact of various restrictions on all agencies. Interorganizational linkages enabled nurturing of relationships among personnel from various agencies. Both shared understanding and interorganizational linkages had been identified previously as key interagency leadership issues with practical implications (40). They allowed for liaison among key stakeholders to be underpinned by mutual respect and constructive conversations, as well as ownership in identifying potential issues and solutions. Advice and practical support were provided as required by various governmental agencies. As a result of this collaboration, there was a synergistic effect of various subject matter expertise coming together in the planning and operationalization of these matches, allowing for various considerations, both specific to COVID-19 (27, 38) and for mass gathering events in general (41), to be addressed. Interagency collaboration for future planning is encouraged both within and outside the context of COVID-19; in South Australia, the nation and beyond.

There is a need for consideration to be given to the potential gap between theoretical recommendations and crowd behavior during sport mega-events (16, 42). During the planning of the Adelaide Oval reopening, this was bridged by ensuring any recommendations were practical and can be easily implemented, with necessary arrangements put in place to encourage such practices. Targeted media and communications prior to the matches contributed to the management of expectations and motivations of the attending spectators (16). Key messages of physical distancing, hygiene and infection prevention and control measures were communicated to attending spectators both in the time leading up to the matches (via targeted communications as well as traditional and social media) and during the matches (via broadcasting of health campaigns and visual reminders including clear signage, ground markings, and visual overlays). Spectators were also discouraged to attend if displaying COVID-19 symptoms, required to provide accurate personal information for contact tracing and encouraged to download the COVIDSafe application. Throughout the AFL and SANFL matches at the Adelaide Oval, adherence to physical distancing, hygiene and infection prevention and control measures was satisfactory, also reflecting the role of personal accountability, responsibility, and solidarity of attending spectators, which had perhaps been understated in the context of COVID-19.

The staged and controlled approach in the reopening of Adelaide Oval and recommencement of sport leagues, with progressively increasing number of maximum spectators (Figure 1), allowed for various measures to be introduced and implemented according to the increasing number of spectators. Measures that worked well continued to be implemented in the next stage, whereas measures that could be improved on were discussed and refined following the match. Some measures that worked in the initial stages also needed to be revisited as the number of spectators increased. For example, as the maximum crowd number approved to attend a sport match increased, the use of alternative turnstiles was replaced with the use of every turnstile to facilitate crowd flow and physical distancing in queues. Spatial considerations also became increasingly important with the increasing number of spectators allowed to attend. These considerations were especially important for queuing areas, waiting areas, bottleneck regions etc. Box 1 summarizes a list of recommendations for the planning of mega-events during COVID-19 and future public health emergencies.

Although there is ongoing debate as to whether elite athletes should be regarded as role models (43–45), elite athletes continue to receive public attention during the COVID-19 pandemic and have the opportunity to influence their followers in either a positive or a negative sense. Although misbehaviors among elite athletes have been prominently reported (45), there was a predominantly positive media report among AFL elite athletes, coaches, and officials. These took the form of positive messaging (encouraging COVID-19 testing, isolation, and quarantine when required) and exemplary, albeit sometimes sacrificial, behavior. To this end, elite athletes and sport league officials could be said to have a role in raising public health awareness and encouraging adherence to health advice. The positive psycho-behavior exhibited by many coaches and elite athletes in the face of a challenging and ever-changing season also has the potential to positively impact on sport fans during and beyond this pandemic. Elite athletes and coaches could be engaged in future partnerships to reinforce public health messaging in community risks and prevention.

The positive impacts of the reopening of Adelaide Oval and the recommencement of sport leagues with spectator attendance were reflected in media reports and were a refreshing change in the largely unbalanced global media landscape in the midst of the pandemic (46). The authors are supportive of the calls for a balanced media reporting of the COVID-19 pandemic and any future outbreaks, including by developing a partnership between public health authorities and the media.

Box 1 **Recommended measures to be considered when planning a mega-event during COVID-19 and future public health emergencies.**

• Collaboration of key stakeholders from various agencies, with close liaison and involvement of public health authorities at all stages of planning and operationalization.
• Local epidemiological context should be taken into account. Where the matches involve attendees of multiple geographical origins, there is additional need for the epidemiological context of these to be considered.
• Planning of mega-events should align with national and local legislation, COVID-19 restrictions and health guidelines.
• Physical distancing measures to be implemented as part of seating arrangements, at queues for admission, point of sales (food and beverages, merchandise etc.), security check points, vertical transport etc.
• Frequent touch points to be regularly cleaned before, during and after mega-events.
• Input from infection prevention and control specialist is encouraged around such measures, including where gloves and masks are used, as incorrect use of these personal protective equipment may be inadequate for infection prevention and control, and provides a false sense of protection.
• Measures implemented should facilitate contact tracing should the need arise, e.g., by allocated seating, collection of personal details upon downloading mobile tickets, etc.
• A staged and controlled increase in maximum number of spectators allows for various measures to be introduced and implemented, with these measures refined as necessary
• Consideration needs to be given to the gap between planning and crowd behavior. Targeted communications prior to mega-events could help manage crowd expectation.
• Personal responsibility of attendees should be emphasized, including by discouraging attendance of people showing COVID-19 symptoms, encouraging attendees to adopt physical distancing, hygiene, infection prevention and control measures.

## Conceptual or Methodological Constraints

The measures put in place as part of the reopening of Adelaide Oval meant that the nature of these matches is quite distinct compared to other mass gatherings, which can promote large influx of people, high degree of social mixing, and proximity through crowded accommodation and associated activities (47, 48). Due to the variable nature of different mass gathering events and the mitigation strategies that can be put in place, as well as the experience from the Adelaide Oval reopening, a case-by-case risk assessment with close involvement of local public health authorities (28) is advocated for proposed mass gathering events during the COVID-19 pandemic and future public health emergencies.

Another challenge of the recommencement of AFL matches at the Adelaide Oval is the multijurisdictional nature of the sport. Comprised of 18 teams from five different Australian states, the recommencement of AFL entailed travel of these teams to and from various jurisdictions, with some teams training and competing in a hub located in another jurisdiction because of varying legislative requirements or varying and changing local epidemiology. While the AFL matches resumed when most jurisdictions experienced low or no active cases, and the attendance of sport spectators at these matches only allowed when assessed to be safe from a public health point of view, there was heightened need for close monitoring of epidemiological and legislative changes in another jurisdiction where AFL matches were also hosted; as well as the movements of individual players, football teams, facilitating personnel and attending spectators. This is consistent with the observation of Parnell et al. (49) with regards to the potentially heightened threat of sustained global spread of COVID-19 as a result of the global sport industry being networked in nature.

South Australia had a prolonged period of no active COVID-19 cases prior to the reopening of Adelaide Oval and recommencement of sport leagues in South Australia. This epidemiological context hugely contributed to the overall very low risk of COVID-19 transmission during sport mega-events in South Australia. This is a very important consideration when our work is used to inform the planning of any other mega-events, especially where mega-events are to be held in a location with a different epidemiological context. There is a need for close liaison between event organizers and other stakeholders with the local public health authorities throughout all stages of the event, including during the planning, during the event and after the event. Furthermore, where a mega-event proceeds following a risk assessment, there is a need for public health authorities and event organizers to continue being mindful of potential transmission especially when the mega-event encourages proximity and contact among attendees.

## Conclusion

The reopening of Adelaide Oval and recommencement of sport leagues with spectator attendance demonstrates that a case-by-case risk assessment can be conducted for mega-events during COVID-19, taking into account the epidemiological context at the time. Some of these mega-events can be safely carried out with appropriate mitigation, including physical distancing, hygiene, and infection prevention and control measures. Where mega-events involve multiple jurisdictions, the epidemiological context of the geographical origins of attendees will also need to be considered. Close liaison and collaboration between event organizers and local public health authorities is strongly recommended throughout the planning and operationalization of mega-events, with a focus on shared understanding and interorganizational linkages. Consideration needs to be given to the potential gap between planning and recommendations and crowd behavior at mega-events, with adequate management of crowd expectation both before and during the event.

Our experience in the reopening of Adelaide Oval and recommencement of AFL and SANFL is satisfactory, with a largely positive impact as reported by the media. No new COVID-19 cases resulted from the reopening of Adelaide Oval and recommencement of sport leagues with spectator attendance.

While much discussion around sports during COVID-19 is the potential for this pandemic to serve as a turning point for the sports industry, including a change in model for sport mega-events (17, 49, 50), one finding around the reopening of Adelaide Oval is that such a proposed change is not easily brought about. At least for the AFL, much effort was put into overcoming the challenges brought about by the pandemic in order to deliver a 2020 season that closely resembles the pre-pandemic model of sport mega-events. Whether an alternative and perhaps more sustainable delivery model for sport mega-events as the rest of the world recovers from COVID-19 remains of interest.

## Data Availability Statement

The original contributions presented in the study are included in the article/Supplementary Material, further inquiries can be directed to the corresponding author/s.

## Ethics Statement

Work in relation to the strategic reopening of Adelaide Oval and recommencement of sport leagues with spectator attendance was undertaken in response to the COVID-19 Public Health Emergency declared in South Australia on March 15, 2020 and a Major Emergency declared on March 22. As such, this work was covered by the *South Australian Public Health Act 2011* and the *Emergency Management Act 2004*. Participation of key stakeholders in this work was voluntary and acknowledged below. This work did not involve any experimental research or identifying data except where publicly available in media reports or websites, as such ethics approval was not required for this publication.

## Author Contributions

All authors contributed to the work around the reopening of the Adelaide Oval and recommencement of sport leagues with spectator attendance. All authors attended the site inspection prior to the reopening of Adelaide Oval and during the Showdown match. AL was involved in the subsequent upskilling of infection prevention and control measures among Adelaide Oval staff. JC wrote the first draft of the manuscript. All authors contributed to manuscript revision, read, and approved the submitted version. An abridged version of this manuscript was submitted to the Australian Health Protection Principal Committee, this was written by NS with input from JC, AL, and CL.

## Conflict of Interest

The authors declare that the research was conducted in the absence of any commercial or financial relationships that could be construed as a potential conflict of interest.

## Publisher's Note

All claims expressed in this article are solely those of the authors and do not necessarily represent those of their affiliated organizations, or those of the publisher, the editors and the reviewers. Any product that may be evaluated in this article, or claim that may be made by its manufacturer, is not guaranteed or endorsed by the publisher.
