# Supplementary material for: Recommencement of Sport Leagues With Spectators at the Adelaide Oval During the COVID-19 Pandemic: Planning, Experience, and Impact of a Globally Unprecedented Approach
Source: Front Public Health. 2021 Jul 23;9:676843. doi: 10.3389/fpubh.2021.676843 (PMC8345120; doi:10.3389/fpubh.2021.676843)
Supplement: Supplementary file 1 [file Data_Sheet_1.DOCX]

Supplementary Material 1 – Mitigating measures for recommencement of sport leagues with spectators at the Adelaide Oval

# Ticketing, seating and contact tracing

AOSMA’s plan for each match included a modelling of seating arrangement for the approved maximum number of spectators. A ballot system was used by both clubs for ticket allocation. Members who were allocated a ticket were required to download their tickets onto their mobile device, following which they were required to enter their contact details and to answer an online declaration form for screening of COVID-19 symptoms. Mobile tickets were scanned at the turnstiles for admission, and where possible, each ticket was downloaded onto a separate mobile device. This ensured accurate records were available for spectators attending the match, should the need for contact tracing arose. Each ticket was allocated a specific seat, allowing for appropriate physical distancing between spectators. Ticketing was configured so that tickets purchased as part of the same transaction were allocated adjacent seats. Each section of the grandstands was staffed by security. Seating arrangements were configured to prevent spectators touching the ball or coming into contact with AFL officials and players. Spectators must be seated at all times.

# Admission, ingress and egress

Two separate entrances into the Adelaide Oval were utilized for the initial match, although one of these entrances has traditionally catered for over 65% of the crowd. This was increased to three entrances as the number of spectators increased. Spectators were encouraged to use the entrance closest to their allocated seats when receiving their tickets. Crowd control barriers were set up to create clear lanes for queues, every alternate lane was blocked off to allow physical distancing between queues. Clear markings spaced 1.5 m apart were available in each lane to allow physical distancing in each queue. Alternative turnstiles were used; this was increased to every turnstile as the number of spectators increased, in order to reduce congestion during ingress. Admission opened several hours before commencement of the game and patrons were encouraged to present early. Alternative access into the Adelaide Oval, for example via its café, were closed off to the public. Clear signage for reminder to practice physical distancing and well-positioned staff were available. At all times security surveillance of the ingress and egress process and back up crowd control measures were also available. Crowd control barriers were removed prior to the end of the match to allow smooth egress. Smaller gates that were not equipped for ingress were also opened for egress.

# Security

Spectators were encouraged not to bring bags. All bags were searched prior to admission. Spectators were asked to place their bags on the table and take a step backwards. Security staff then stepped towards the table and conducted the bag search without touching the bags or belongings in the bags. Security staff were provided with extendable wands for random checks. Such checks were conducted with spectators and security staff in an off-centered position and as much distance as possible.

# Vertical transport

A staff member was positioned at each escalator, who reminded spectators to leave two steps before getting onto the escalator. All escalators were programmed for the same direction to facilitate crowd flow, i.e. all escalators were programmed to go upwards during ingress, and to go downwards during egress. Handrails of escalators were regularly cleaned. Lifts were limited to four people at a time, and were available for disabled spectators and spectators hosted in function rooms. Lifts were also cleaned on a regular basis throughout the night.

# Toilets

Toilets at the Adelaide Oval were designed with separate entrances and exits, providing a one-way traffic for spectators. Each toilet was staffed at its entrance to limit the number of spectators using each toilet at one time. Every second urinal and cubical was used. Most toilets at the Adelaide Oval do not have doors, and are equipped with censor-activated soap dispensers and taps; reducing touch points for fomite transmission of COVID-19. Regular cleaning of toilets occurred during and after the match.

# Food and beverage

Spectators from each section were encouraged to stay in a section of the stadium. Each section was served by separate food and beverage point of sales, limiting the intermixing of patrons seated in separate stands. Clear ground markings and bollards were available at each point of sales for management of crowd flow and physical distancing. Pre-packaged food was served. Food and beverage were to be consumed while seated only.

# Public transport

AOSMA works closely with the Department for Infrastructure and Transport with regards to public transport and effective management of spectators arriving via public transport. For matches with less than 10,000 spectators, regular public transport was maintained with no additional overlay. For matches with more than 10,000 spectators, additional overlay was provided. Spectators were reminded that buses will not leave until the game is over or maximum capacity (accounting for physical distancing in buses) is reached. Additional route of access was also opened up to facilitate crowd flow to the train station. Electronic boards were used to remind people of physical distancing especially during ingress and egress. SAPOL presence was made available at converging points. Major roads around the Adelaide Oval were closed for matches with high number of spectators. All staffing and resources, both within the Adelaide Oval and in the surrounding, were planned according to the full event capacity of the Adelaide Oval. Major converging points were monitored for crowding with SAPOL presence.

# Car parks

One of the two car parks at the Adelaide Oval was closed off to the public and accessible to AFL officials and players only. AFL officials and players were able to enter the Adelaide Oval from this car park and to proceed to change rooms without interaction with any other persons. The other car park was open for Adelaide Oval staff and spectators. Payment could be made using cards or non-touch payments only.

# Media and communications

Targeted communications with key messages had a key role in managing the expectations of sport spectators. Spectators were sent a detailed email including physical distancing guidelines two days prior to the match. A further text message was sent to each patron three hours prior to the match. A media conference was held six hours prior to the match, involving the Minister for Health and Wellbeing, Chief Public Health Officer and Chief Executive Officer of AOSMA. The media conference included footage of crowd control barriers and ground markings for physical distancing at the entrance. SA Health social media also reminded spectators of the key messages for the match following the media conference. Spectators were encouraged to download the COVIDSafe application, which was developed as a national tool to facilitate contact tracing. The AOSMA also included reminder slides for physical distancing and COVIDSafe application; as well as SA Health campaigns for COVID-19. All visual backups were overlaid with reminder for physical distancing.

# Sponsor handouts and merchandise

There were no sponsor handouts and sales of merchandise for the initial Showdown match. Principles outlined for food and beverage above were applied to merchandise sales as these were made available at matches.

# Function rooms

Maximum number of spectators hosted in function rooms complied with restrictions and health guidelines at the time. Dining and drinking were permitted when seated only. Each table was set up for two to four spectators. Each function room was serviced by its own kitchen. Meals were served on individual plates.

# Change rooms

Each football club was allocated two change rooms. These were managed by individual clubs in accordance to the AFL COVID-19 protocol.

# Live broadcast

Spectators who were not allocated a ticket were able to watch the live broadcast either from home or from the drive-in cinemas.
