# Supplementary material for: Recommencement of Sport Leagues With Spectators at the Adelaide Oval During the COVID-19 Pandemic: Planning, Experience, and Impact of a Globally Unprecedented Approach
Source: Front Public Health. 2021 Jul 23;9:676843. doi: 10.3389/fpubh.2021.676843 (PMC8345120; doi:10.3389/fpubh.2021.676843)
Supplement: Supplementary file 2 [file Data_Sheet_2.DOCX]

Supplementary Material 2 – Impact of COVID-19, Reopening of Adelaide Oval and Recommencement of Sport Leagues with Spectator Attendance

The impacts of COVID-19, reopening of Adelaide Oval and recommencement of sport leagues, both economic and non-economic, were documented by means of qualitative analyses of media reports and website releases between March 1 and July 27, 2020. The dates represented two weeks prior to the commencement of the 2020 AFL season, and one week after the AFL match with an approved maximum of 25,000 spectators.

There were far-reaching impacts of COVID-19 on sport leagues, elite athletes and their family, staff of football clubs, league officials, staff of Adelaide Oval, sport spectators and the South Australian community. When announcing the postponement of its 2020 season, the AFL chief executive officer stated that

*…to say this is the most serious threat to our game in 100 years is an understatement… It is unprecedented in the impact it is having on our game and the wider community…* (Barrett, 2020b)

While the most obvious impact of COVID-19 on the Australian football leagues and their football clubs was the interruption of their 2020 seasons, the consequences of the interruption and the subsequent effort in recommencing the seasons are more elusive.

# Economic impacts

The economic impacts of COVID-19 due to interruption of the AFL season had been a focus of media reports, even though not formally quantified at time of writing. One club expressed that

*There is no sugar coating what the potential financial implications of the decision to postpone the AFL season are, and what that means for the future… Our survival is at stake.* (Fox Sports, 2020b)

The AFL alone was considered to provide “livelihoods for thousands and thousands of people”, many of whom were stood down during the pandemic, including staff of the league, football clubs and hosting venues (Barrett, 2020b). League officials, coaches and elite athletes also took pay cuts during the interrupted season, although different groups were likely to be impacted financially to different extents. For example, many staff of the Adelaide Oval only worked during major events to supplement a primary income, therefore did not qualify for national welfare payments through the Adelaide Oval.

While refund options for club memberships were made available, both AFL and its clubs implored members for their ongoing support during COVID-19:

*If they need it, they can, but yes clearly we’d love them to stay because our industry is in a battle and our clubs are in a battle to get through and the membership is their lifeblood… if they’re able to maintain their membership and keep going, it will go a long way to getting their clubs through this…* (Murphy, 2020)

*If you believe that the clubs and AFL football is important, please stick with us at the moment… if you don’t have members… if we don’t have enough people putting their hand in their pocket, there will be no club…* (De Silva, 2020)

Even with the recommencement of its season, various elements introduced in response to COVID-19 also resulted in unbudgeted expenses. For example, enhanced COVID-19 testing of all players and staff costs $100 each time a swab is taken. The shortened season and shortened quarters also had implications on broadcasting revenues. Reportedly, each round of the AFL matches was costing the league between $20-25 million (Landsberger and Williams, 2020). The ongoing considerations of the economic impacts following the recommencement of AFL were typified in comments such as

*This is financially challenging for the industry and we’re making obviously tough decisions.* (Larkin, 2020)

*AFL stood firm today… said “we’re not going to fork out the cash for the charter flights”* (7News Adelaide, 2020e)

*It would be much better for us to have … short term pain but be able to continue… rather than be stuck at home… without a salary. That would’ve been a bigger price to pay…* (7News Adelaide, 2020b)

Indeed, the economic revenue of AFL had been well documented to surpass many other sports in Australia, both as a direct result of ticket sales, memberships, merchandise etc. as well as indirect profit from interstate travel, accommodations, hospitality and entertainment (Stewart, 2014). Indirect revenue generated during a sport mega-event is estimated to be up to 2.5 times that of direct revenue. (Sports Venue Business, 2020). Much of the activities that generate both direct and indirect revenues were prevented during the 2020 AFL season, both due to restrictions impacting on the season, such as the need for matches to proceed with no or limited number of spectators, as well as restrictions impacting on industries such as hospitality and entertainment. This was also reflected in various industries pleading for the restrictions around their industries to be relaxed following the approval of spectator attendance at the AFL Showdown match.

### On the other hand, alternative arrangements to facilitate the recommencement of the 2020 AFL season could have mitigated some economic impacts of COVID-19 to certain businesses and regions, though at a cost of the AFL. For example, where local COVID-19 epidemiology or governmental restrictions required football teams to be relocated to another jurisdiction, football teams were accommodated in hospitality facilities that were impacted economically due to a reduction in travel (Walsh, 2020). The relocation of ten Victorian teams to Queensland amid a spike in COVID-19 cases was estimated to cost at least $3 million a week (Larkin, 2020). During this time, the AFL also secured line of credits from banks.

# Psychobehavioral impacts

## Focus and positivity amidst challenges

With the arrangements of matches dependent on the evolution of local epidemiology, there was much uncertainties throughout the AFL season following its recommencement. Driven by the determination to complete the 2020 season, these uncertainties necessitated quick decision making and flexibility in response to rapid changes in the local COVID-19 situation and restrictions, each presenting with unique challenges. In the words of the AFL chief executive officer,

*…this would be a season of challenges. And that the one certainty is that before we complete the season there would be more unique challenges along the way.*

*We know as this situation continues to evolve, we have to remain agile and flexible to be able to adapt where necessary…*

As various alternative arrangements were put in place to facilitate the AFL season, there was a need for clubs and venues involved to be resilient and flexible. Some of these arrangements were a significant departure from what elite athletes, football clubs and venues were accustomed to, as could be seen in the comments of a football coach,

*There’s been a lot of distractions for these players… protocols, travel, away from home, can’t go to the shops, can’t do extra training… we’re doing whatever we can to work within those…* (7News Adelaide, 2020a)

It was clear that the ability to remain focused on the goal amidst these uncertainties became an advantage for football clubs and elite athletes. Following the need for another football club to relocate within short notice, its football coach responded saying,

*…we’re going up there with some certainty in our own mind that we’re going up there for three games… and to play well in those three games. So we’re not going to get distracted by anything other than we’re preparing to play footy…* (Valencich, 2020)

The AFL chief executive officer went a step further in saying

*I fundamentally believe that the team that wins this year’s flag will be the team that accepts the change, races and challenge and shows a resilience to get on…* (7News Adelaide, 2020c)

## Fostering of community and collaboration

These uncertainties also encouraged community within football clubs and collaboration within the football industry, in addition to the interagency collaboration in facilitating the reopening of Adelaide Oval and recommencement of sport leagues. One coach commented before the club returned to their state of residence that

*It will be good to see family… but at the same time we quite enjoyed being up here… we’ll stick together…* (7News Adelaide, 2020a)

Coordinating the logistics of relocating football clubs and ensuring compliance to AFL’s strict COVID-19 protocols, an AFL general manager stated that

*We might have multiple changes daily, and it is challenging, but… It’s really a credit to all the clubs and the industry working together… I’ve been working in the AFL for fifteen years and this is probably the time where we’ve had the best communication and relationship with our clubs in terms of solving the challenges ahead of us.* (Walsh, 2020)

## Tensions with conflicting interests and priorities

However, these uncertainties and the subsequent arrangements, many of which implemented with short notice, were sometimes challenged as a result of conflicting interests and priorities. One such conflict is the impact of these arrangements on families of elite athletes, staff and league officials. During the worsening COVID-19 outbreak in Victoria, the relocation of ten football teams was initially expected to last for around thirty-five days, following which this was revised to at least nine weeks. The captain of a football club was the first to voice that such arrangements could be difficult, given that

*We’ve got guys with pending baby and other challenges or things going on in their life…* (7News Adelaide, 2020b)

The AFL Players Association also advocated for ongoing payment of players who were unable to relocate for family reasons (Fox Sports, 2020a); whereas some coaches were less receptive of elite athletes who opt out of such alternative arrangements, with one coach stating that he

*Wouldn’t trust him in the team again… the legacy you leave behind is what you do in football and having done that you can look after your family… It’s as simple as that…* (Zita and Fox Sports, 2020)

Subsequently, AFL acknowledged the sacrifice of families of elite athletes and league officials in facilitating the recommencement of the AFL season, and made arrangements for families to be accommodated with elite athletes and league officials who had to relocate to another jurisdiction (Barrett, 2020a). Support was also provided for elite athletes who had to return to their home state due to unforeseen circumstances (McFarlane and Herald Sun, 2020).

# Impacts on public health awareness

COVID-19, the interruption of the AFL season and the subsequent recommencement also had impacts on the public health awareness of the community. During this time, much of the media releases by AFL and the football clubs continued to reinforce key public health messages:

*We tried our best as an industry to try to forge ahead but now it’s time for us to close our doors for a period of time and do what everyone else needs to do – and that’s get isolated and stay safe.* (Fox Sports, 2020b)

*We are clearly in uncharted territory and not just as an industry, but as a society… The health of all Australians is the priority and we will continue to listen to the advice of governments and medical experts…* (Thomas-Wilson et al., 2020)

Elite athletes and their families who contracted COVID-19 also promoted awareness on the disease and encouraged public health measures including getting tested, complying with requirements for isolation and quarantine, etc.

*My family has been affected by COVID-19…my mother… she is currently in ICU… think about it… as it being you and part of your family, and hopefully it can change your mindset about COVID-19. The reality is it is out there… I am experiencing it right now…* (Williams et al., 2020)

*Go get tested, do your bit… I’ve been tested over thirty times being an AFL player… it tickles a little bit it doesn’t hurt… and do your best to isolate, and we do our best to wipe out this pandemic.* (Williams et al., 2020)

AFL also had strict COVID-19 protocols to facilitate the recommencement of its 2020 season. The protocols received input from public health authorities of all Australian jurisdictions involved, and included regular COVID-19 testing, minimizing unnecessary contact between elite athletes and staff with the general population, rules around length and type of training etc. These protocols, as well as any consequential outcomes (such as elite athletes testing positive for COVID-19 or any breaches of the protocol), received considerable media attention, and reinforced to the public the public health principles that needed to be adhered to.

# Community benefits

## Community cohesion

Community cohesion is one of the most prominent themes reported in the media following the reopening of the Adelaide Oval for the recommencement of the 2020 AFL season and the commencement of the SANFL season. The positive media narrative is typified in statements including

*Fans were three seats apart yet they’ve never been closer, they knew they were sharing something special, grateful to be among the lucky 2000.* (7News Adelaide, 2020h)

*It couldn’t have been a more spectacular winter’s day for the return for SANFL footy, even fans on the losing side just seem to be enjoying their weekend ritual with their mates at the footy* (7News Adelaide, 2020f)

While the use of artificial crowd noise had been explored in other settings, the atmosphere at the Adelaide Oval with only two thousand spectators on the grandstands during the AFL Showdown match was no inferior compared to previous AFL matches. Indeed, crowd noise had been said to play an important role in bringing about the sense of belonging and bonding among sport spectators (Russell, 2020). Interviewed just prior to entering the Adelaide Oval, one Port Adelaide Football Club fan stated that

*Fourteen hundred Port supporters can make enough noise to fill the stadium…* (9 News Adelaide, 2020)

Another football fan commented after the Showdown match that the Adelaide Oval felt like

*…a small country ground, there was this real sense of belonging, we were all the true believers…* (7News Adelaide, 2020h)

## Community pride, visibility and image

Another prominent theme in media reports following the reopening of Adelaide Oval and the first sporting event with crowds is the community pride of this success. The Advertiser published a souvenir poster calling the night ‘A Showdown like no other’. The positive media narrative is captured in reports such as

*We’ve never witnessed scenes quite like this… this was one for the history books… a Showdown in the midst of a pandemic.* (7News Adelaide, 2020h)

The media, AOSMA and sport spectators were well aware of South Australia being the focus of the nation and beyond on the night of the Showdown match, as can be seen in these comments

*It’s a privilege… Hopefully we can make an example for more events to have crowds… (9* News Adelaide, 2020)

*The eyes of the nation are watching Adelaide Oval tonight to see how it all unfolds… Just a few weeks ago it all seems so unlikely.* (7News Adelaide, 2020g)

*Our job we know is to represent South Australia, really on a world stage. We have a lot of stadia around the world looking at what our procedures are…* (7News Adelaide, 2020d)

There was also considerable media attention on Eleni Glouftsis, a South Australian football umpire who became the first female umpire to officiate a Showdown match between Adelaide Football Club and Port Adelaide Football Club. Earle (2020) described it in The Advertiser as follows

*SA umpire Eleni Glouftsis will make a sacrifice to officiate at the Showdown – and make history as well. Having become the AFL’s first female field umpire in 2017 aged 25 and Young South Australian of the year in 2019, Glouftsis is now realising a cherished Showdown ambition.*

# References

7news Adelaide (2020a). *Adelaide Crows coach Matthew Nicks on new explosive details from the 2018 Gold Coast camp saga* [Online]. Available: <https://www.facebook.com/7NEWSAdelaide/videos/611886256113010/?v=611886256113010> [Accessed July 15, 2020].

7news Adelaide (2020b). *AFL fixture chaos due to border restrictions* [Online]. Available: <https://www.facebook.com/watch/?v=2943731262392101> [Accessed July 15, 2020].

7news Adelaide (2020c). *AFL season in turmoil* [Online]. Available: <https://www.facebook.com/7NEWSAdelaide/videos/1164907590551561/?v=1164907590551561> [Accessed July 15, 2020].

7news Adelaide (2020d). *Crowds at Adelaide Oval* [Online]. Available: <https://www.facebook.com/watch/live/?v=1400806993451380> [Accessed July 15, 2020].

7news Adelaide (2020e). *Double blow for the Crows as Port Adelaide prepares for GWS* [Online]. Available: <https://www.facebook.com/watch/?v=747147722701478> [Accessed July 15, 2020].

7news Adelaide (2020f). *Footy fans rejoice as SANFL returns at Adelaide Oval* [Online]. Available: <https://www.facebook.com/watch/?v=598669301087407> [Accessed July 16, 2020].

7news Adelaide (2020g). *Historic Showdown 48 to get underway at Adelaide Oval* [Online]. Available: <https://www.facebook.com/watch/?v=1169044083442945> [Accessed July 15, 2020].

7news Adelaide (2020h). *More fans could be allowed in stadiums as Showdown declared roaring success* [Online]. Available: <https://www.facebook.com/watch/?v=2585153361584294> [Accessed July 16, 2020].

9 News Adelaide (2020). *Historic Showdown* [Online]. Available: <https://www.facebook.com/watch/?v=183976439692704> [Accessed June 14, 2020].

Barrett, D. (2020a). *Fixture bonanza: Get set for 19 straight days of footy* [Online]. Available: <https://www.afl.com.au/news/465498/fixture-bonanza-get-set-for-19-straight-days-of-footy> [Accessed].

Barrett, D. (2020b). *'Most serious threat in 100 years': AFL postpones season* [Online]. Available: <https://www.afl.com.au/news/389109/-most-serious-threat-in-100-years-afl-postpones-season> [Accessed July 16, 2020].

De Silva, C. (2020). Collingwood president Eddie McGuire and Tony Jones in fiery exchange over AFL membership refunds. *Nine*.

Earle, R. (2020). Eleni Glouftsis says she’s honoured to do her bit for football and quarantine before becoming first woman to officiate a Showdown. *The Advertiser*.

Fox Sports (2020a). AFLPA expects players to be paid even if they opt out of interstate hubs.

Fox Sports (2020b). As the AFL braces for a time of immense uncertainty, clubs have delivered supporters these messages.

Landsberger, S., and Williams, R. (2020). Hubs take heavy toll on AFL bottom line, hence push for all 153 games to be played. *Herald Sun*.

Larkin, S. (2020). AFL moves Victorian clubs into Queensland. *7News*.

Mcfarlane, L., and Herald Sun (2020). Gary Ablett leaves AFL hub to return home to Victoria to be with family. *The Advertiser*.

Murphy, C. (2020). AFL boss confirms membership refunds available during coronavirus crisis after Eddie McGuire and Tony Jones clash on live TV. *ABC News*.

Russell, A. (2020). Why does crowd noise matter? *The Conversation*, p.July 16, 2020.

Sports Venue Business (2020). *Coronavirus’ economic impact on the sports industry* [Online]. Available: <https://sportsvenuebusiness.com/index.php/2020/03/19/coronavirus-economic-impact-on-the-sports-industry/> [Accessed July 1, 2020].

Stewart, B. (2014). Why AFL grand final is the most successful event in Australia. *The Conversation*.

Thomas-Wilson, S., Warner, M., Horne, B., Wood, L., and Herald Sun (2020). South Australia will not permit Victorian AFL teams to hub in Adelaide due to the risk of them bringing COVID-19 into the state. *The Advertiser*.

Valencich, G. (2020). South Australia clears Port Adelaide and Adelaide to leave AFL hub on the Gold Coast. *7News*.

Walsh, L. (2020). What life is really like for AFL teams currently living inside interstate hubs for 2020 season. *The Advertiser*.

Williams, R., Wood, L., Landsberger, S., Thomas-Wilson, S., and Herald Sun (2020). AFL unveils Round 8 fixture as Victorian clubs brace for longer periods away from home. *The Advertiser*.

Zita, D., and Fox Sports (2020). ‘Wouldn’t trust him in the team again’: AFL great’s brutal response to reluctant hub players.
