# Supplementary material for: Recommencement of Sport Leagues With Spectators at the Adelaide Oval During the COVID-19 Pandemic: Planning, Experience, and Impact of a Globally Unprecedented Approach
Source: Front Public Health. 2021 Jul 23;9:676843. doi: 10.3389/fpubh.2021.676843 (PMC8345120; doi:10.3389/fpubh.2021.676843)
Supplement: Supplementary file 3 [file Table_1.DOCX]

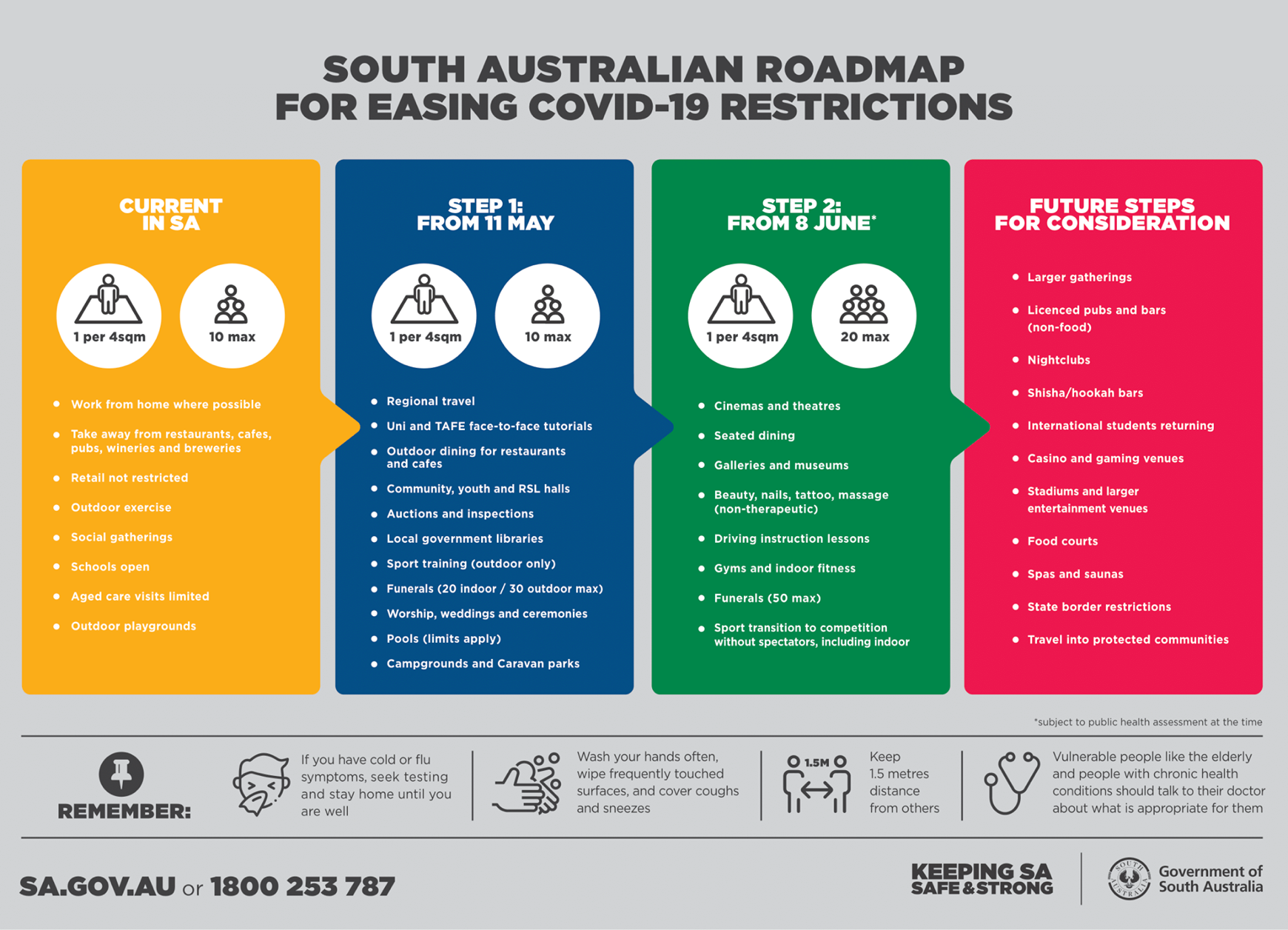


**Supplementary Figure 1.** The recommencement of sport activities and sport leagues were included in the South Australian Roadmap for Easing COVID-19 Restrictions, released 8 May 2020 and updated subsequently as required.
